# SARS-CoV-2 and ORF3a: Nonsynonymous Mutations, Functional Domains, and Viral Pathogenesis

**DOI:** 10.1128/mSystems.00266-20

**Published:** 2020-05-05

**Authors:** Elio Issa, Georgi Merhi, Balig Panossian, Tamara Salloum, Sima Tokajian

**Affiliations:** a Department of Natural Sciences, School of Arts and Sciences, Lebanese American University, Byblos, Lebanon; University of California San Diego

**Keywords:** 3a protein, COVID-19, nonsynonymous mutations, ORF3a, SARS-CoV-2

## Abstract

The effect of the rapid accumulation of nonsynonymous mutations on the pathogenesis of severe acute respiratory syndrome coronavirus 2 (SARS-CoV-2) is not yet known. The 3a protein is unique to SARS-CoV and is essential for disease pathogenesis. Our study aimed at determining the nonsynonymous mutations in the 3a protein in SARS-CoV-2 and determining and characterizing the protein’s structure and spatial orientation in comparison to those of 3a in SARS-CoV. A total of 51 different nonsynonymous amino acid substitutions were detected in the 3a proteins among 2,782 SARS-CoV-2 strains. We observed microclonality within the ORF3a gene tree defined by nonsynonymous mutations separating the isolates into distinct subpopulations. We detected and identified six functional domains (I to VI) in the SARS-CoV-2 3a protein. The functional domains were linked to virulence, infectivity, ion channel formation, and virus release. Our study showed the importance of conserved functional domains across the species barrier and revealed the possible role of the 3a protein in the viral life cycle. Observations reported in this study merit experimental confirmation.

**IMPORTANCE** At the surge of the coronavirus disease 2019 (COVID-19) pandemic, we detected and identified six functional domains (I to VI) in the SARS-CoV-2 3a protein. Our analysis showed that the functional domains were linked to virulence, infectivity, ion channel formation, and virus release in SARS-CoV-2 3a. Our study also revealed the functional importance of conserved domains across the species barrier. Observations reported in this study merit experimental confirmation.

## Abstract (precis)

At the surge of the coronavirus disease 2019 (COVID-19) pandemic, we detected and identified six functional domains (I to VI) in the SARS-CoV-2 3a protein. Our analysis showed that the functional domains were linked to virulence, infectivity, ion channel formation, and virus release in SARS-CoV-2 3a. Our study also revealed the functional importance of conserved domains across the species barrier. Observations reported in this study merit experimental confirmation.

## OBSERVATION

The rapid spread of coronavirus (CoV) disease 2019 (COVID-19), caused by severe acute respiratory syndrome CoV 2 (SARS-CoV-2), caused a major global concern (1). Coronaviruses are enveloped positive-sense RNA viruses and are broadly distributed in humans and mammals. The genome of SARS-CoV-2 showed 96.2% sequence similarity to a bat SARS-related coronavirus (SARS-CoV RaTG13) collected in Yunnan Province, China (1), and 79% and 50% similarities to SARS-CoV and Middle East respiratory syndrome CoV (MERS-CoV), respectively (2). A 91% similarity to pangolin CoV suggested that pangolins can be considered possible hosts in the emergence of the novel coronavirus (3). The 3a protein (NCBI accession number YP_009724391.1) showed 72% sequence similarity to that detected in SARS-CoV (4).

We investigated the presence in SARS-CoV-2 of functional domains in the 3a protein linked to virulence, infectivity, ion channel formation, and virus release. We then studied the diverse nonsynonymous mutations in ORF3a and investigated the effect of newly introduced mutations in the localization and tree topology of the 3a protein in SARS-CoV-2.

## 

### Microclonality within ORF3a.

**FIG 1:**
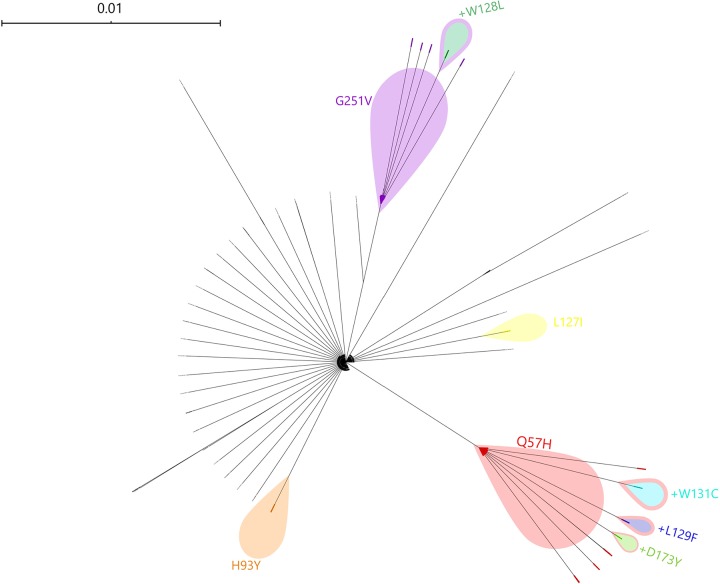
Phylogenetic tree of a SARS-CoV-2 ORF3a gene tree highlighting microclades with nonsynonymous deleterious mutations.

Signature mutations within SARS-CoV-2 ORF3a cause the isolates to cluster into defined phylogenetic clades (Fig. 1). We observed microclonality within the ORF3a gene tree defined by the nonsynonymous mutations separating the isolates into distinct subpopulations, highlighted in Fig. 1. Moreover, three isolates of the Q57H clade with the Q57H mutation were identified to contain second mutations: D173Y (EPI_ISL_419177), W131C (EPI_ISL_418188), and L129F (EPI_ISL_418241). One isolate, namely, EPI_ISL_411929 from the G251V clade, also had a W128L mutation.

### Nonsynonymous mutations in SARS-CoV-2 ORF3a.

The 3a protein showed a 97.82% sequence similarity to a nonstructural protein, NS3, of bat coronavirus RaTG13 (NCBI accession number QHR63301.1). The alignment of the ORF3a protein sequences extracted from the 2,782 available genomes revealed in total 51 different nonsynonymous amino acid (aa) substitutions (Table 1). Q57H and G251V were the most common and identified in 17.43% (*n* = 485) and 9.71% (*n* = 270) of the genomes, respectively.

**TABLE 1:** List of 51 nonsynonymous amino acid substitutions in ORF3a among 2,782 strains

Amino acids substitution in ORF3a^*a*^	Incidence^*b*^	PROVEAN score	Variation effect on protein^*c*^
F8L	1	–4.943	Deleterious
G11V	1	–8.667	Deleterious
V13L	8	–1.648	Neutral
T14I	7	–4.61	Deleterious
S26P	1	–0.981	Neutral
A31T	2	–1.295	Neutral
T34M	1	–1.714	Neutral
G44V	1	–5.533	Deleterious
L46F	1	–3.295	Deleterious
G49C	1	–6.581	Deleterious
A54V	2	–2.295	Neutral
F56C	1	–6.257	Deleterious
**Q57H**	485	–3.286	Deleterious
K61N	3	–3.286	Deleterious
K67N	2	–1.029	Neutral
K75E	2	–0.962	Neutral
G76S	1	0.057	Neutral
V88A	2	–2.962	Deleterious
V88L	1	0.029	Neutral
T89I	1	–4.943	Deleterious
**H93Y**	14	–3.943	Deleterious
A99V	23	–1.962	Neutral
G100C	1	–4.781	Deleterious
G100V	1	–4.829	Deleterious
P104H	1	–3.676	Deleterious
M125I	1	–0.59	Neutral
**L127I**	1	–0.667	Neutral
**W128L**	1	–7.752	Deleterious
**L129F**	1	–3.829	Deleterious
**W131C**	1	–7.752	Deleterious
L140V	2	–0.943	Neutral
C153Y	1	–0.248	Neutral
D155Y	1	–6.829	Deleterious
G172C	1	–6.752	Deleterious
**D173Y**	1	–6.495	Deleterious
T175I	3	2.562	Neutral
T176I	1	–4	Deleterious
Y189C	11	–7.581	Deleterious
E191G	1	–4.933	Deleterious
G196V	45	–6.581	Deleterious
S205T	1	0.019	Neutral
G224C	1	–7.581	Deleterious
G224V	1	–8.914	Deleterious
V225F	1	–2.876	Deleterious
Q245P	1	–4.943	Deleterious
**G251V**	270	–8.581	Deleterious
G251C	1	–8.914	Deleterious
S253F	1	–3.276	Deleterious
G254R	3	–5.257	Deleterious
V259L	1	–0.657	Neutral
T269M	2	–2.381	Neutral

a Mutations analyzed herein are shown in bold.

b Percentage values in this column do not add up to 100%, as mutations cover only a fraction of the total sample size. The total number of sequences was 2,782.

c The cutoff value was −2.5.

### Functional domains.

**FIG 2:**
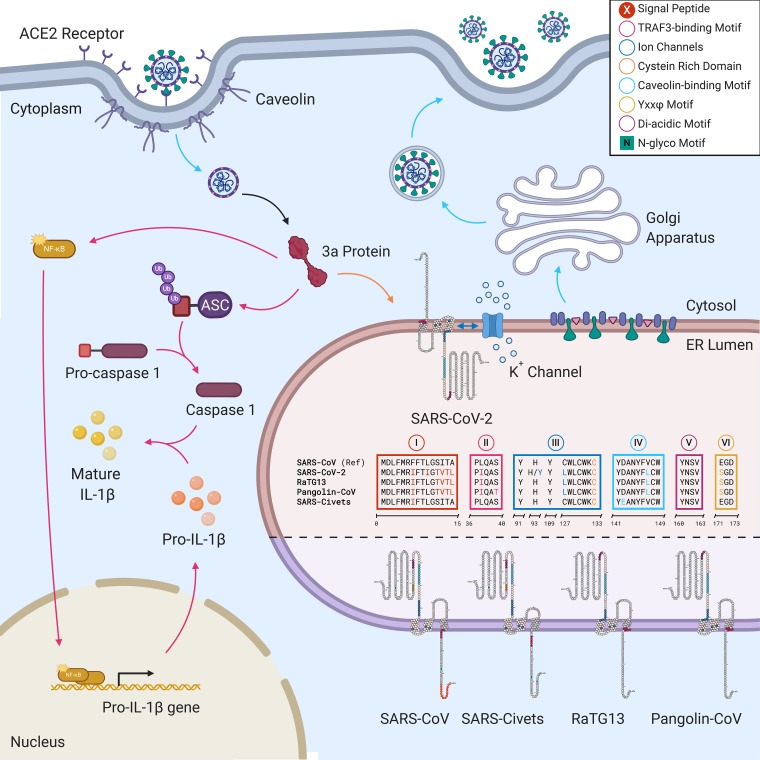
Schematic representation of the hypothetical pathway of the 3a protein function, including a comparison of functional domain sequences and membrane topology in the 3a protein. Arrows are color-coded according to the functional domains involved (top right key); the 3a protein structure (in red) is illustrated by a generic protein icon (not scaled to a three-dimensional structure). ER, endoplasmic reticulum; IL-1β, interleukin 1β; Ub, ubiquitin. Created by BioRender.

We divided the 3a protein into six functional domains (I to VI) based on previously reported data and color-coded each domain for its role within the host cell (see Table S1 in the supplemental material and Fig. 2). Then, we aligned and compared the amino acid sequences in SARS-CoV (NCBI accession number P59632), SARS-CoV-2 (UniProtKB accession number P0DTC3/NCBI accession number YP_009724391), RaTG13 (EPI_ISL_402131), pangolin CoV (EPI_ISL_410721), and civet SARS (NCBI accession number AAU04650.1) to determine whether or not SARS-CoV-2 has similar functional domains and to accordingly follow and determine whether any of the introduced nonsynonymous mutations has a potential impact on the virus’ virulence and pathogenesis.

10.1128/mSystems.00266-20.1 TABLE S1 Protein domains in the 3a protein of SARS-CoV (NCBI accession number P59632) were compared to those of the 3a proteins in SARS-CoV-2 (UniProtKB accession number P0DTC3), RaTG13 (NCBI accession number QHR63301.1), pangolin CoV (EpiCoV accession number EPI_ISL_410721), and civet SARS (NCBI accession number AAU04650.1). Six protein domains (I to VI) were identified. Domain I (not shown) includes a 15-aa signal peptide. Download Table S1, PDF file, 0.1 MB. Copyright © 2020 Issa et al. 2020 Issa et al. This content is distributed under the terms of the Creative Commons Attribution 4.0 International license.

One of the immediately observed differences was the absence of the previously detected N terminus putative signal peptide (Fig. 2, aa 1 to 15, domain I) in SARS-CoV (Table S1), confirmed using Protter v1.0 (5), from all the other studied strains, including SARS-CoV-2 (domain I). The 3a protein in SARS-CoV-2 and all other studied strains, as with SARS-CoV, had three transmembrane regions (Fig. 2).

Domain II contained the TRAF3-binding motif in SARS-CoV, which was also detected in SARS-CoV-2. We observed in domain II of SARS-CoV-2 two amino acid substitutions (positions 36 and 40; amino acid substitutions are shown in bold in the motifs below). A PLQAS motif was conserved in SARS-CoV and civet SARS in domain II, while the L37I substitution (P**I**QAS motif) was detected in SARS-CoV-2 and RaTG13, with the pangolin CoV additionally having an S40T substitution (P**I**QA**T** motif) (Fig. 2; Table S1).

Domain III consisted of a K^+^ ion channel (positions 91 to 133) and a cysteine-rich domain (positions 81 to 160) in SARS-CoV. We noticed that in this domain, Y91 and Y109 were conserved (Fig. 2). Several mutations were identified within this domain in SARS-CoV-2 and included H93Y, L127I, W128L, L129F, and W131C. The sequence alignment of 2,782 SARS-CoV-2 3a proteins revealed a 0.5% (*n* = 14) prevalence of H93Y (source, Wales, UK; date, 12 March 2020 to 20 March 2020) and a 0.036% (*n* = 1) prevalence of L127I (EPI_ISL_418264; source, Greece; date, 18 March 2020), W128L (EPI_ISL_411929; source, South Korea; date, January 2020), L129F (EPI_ISL_418241; source, Algeria; date, 02 March 2020), and W131C substitutions. The W131C mutation detected in strain EPI_ISL_418188 (source, USA; date, 23 March 2020) added a third cysteine residue to this domain in SARS-CoV-2.

A cysteine-rich region was also observed between positions 81 and 160. Cysteine residues were previously reported as being involved in the homodimerization of the 3a protein in SARS-CoV (6). The most important residue for homodimerization was C133 and was conserved in all studied strains (Fig. 2, domain III).

Domain IV consisted of a caveolin-binding motif (Fig. 2, positions 141 to 149; Table S1). A single amino acid substitution was observed in SARS-CoV-2, RATG13, pangolin CoV (YDANYF**L**CW motif), and civet SARS (Y**E**ANYFVCW motif).

The YXXΦ motif was detected in all studied strains, including SARS-CoV-2 in domain V (motif, YNSV; positions 160 to 163). Finally, domain VI in SARS-CoV consisted of a diacidic motif, ExD, at positions 171 to 173 (Table S1; Fig. 2). The diacidic EGD motif was conserved in SARS-CoV and civet SARS, while E171S changed the motif to SGD in SARS-CoV-2, RaTG13, and pangolin CoV. A D173Y substitution was detected in one SARS-CoV-2 strain (EPI_ISL_419177; source, France; date, 22 March 2020), completely disrupting the diacidic motif.

ORF3a encodes a minor structural protein of 274 aa residues in SARS-CoV (7). In this study, we divided the 3a protein into six functional domains (I to VI) based on previously reported data from SARS-CoV and color-coded each domain for its role within the host cell (Fig. 2).

We linked the TRAF3-binding motif in SARS-CoV to domain II and found that we have a similar one in SARS-CoV-2. The 3a protein in SARS-CoV, associated with TRAF3 through the TRAF3-binding motif, was found to activate NF-κB and the NLRP3 inflammasome (8).

Domain III had the K^+^ ion channel and cysteine-rich domain in SARS-CoV (6, 9). We observed several mutations within this domain in SARS-CoV-2. H93Y was particularly important, previously being linked in SARS-CoV to the loss of the K^+^ channel and reduced proapoptotic activity (9). A cysteine-rich region between positions 81 and 160 was also detected in SARS-CoV-2. 3a in SARS-CoV forms interchain disulfide bonds on the interior side of the viral envelope with the spike (S) protein though cysteine-rich regions, and the biological function of the 3a protein was correlated with that of the S protein in SARS-CoV (7).

Additionally, cysteine residues were associated with the homodimerization of the 3a protein in SARS-Co. C133 was particularly important in maintaining the homodimer (6), which was conserved in all viruses, including SARS-CoV-2.

Domain IV consisted of a caveolin-binding motif in SARS-CoV (10). Potential interactions with caveolin-1 may regulate the uptake and trafficking of the 3a protein to the plasma or endomembranes (10).

In all the studied strains, the conserved YXXΦ motif in domain V, which had a significant role in the transport of the 3a protein from the Golgi apparatus to the plasma membrane in SARS-CoV (11), was another important finding. Mutations in this motif were linked to the aggregation of the 3a protein in the Golgi apparatus. Maintaining the YXXΦ motif in all strains confirms its role in 3a intracellular trafficking and surface transport, which otherwise would be targeted to lysosomal degradation via lipid droplets (11). A diacidic motif on the C terminus of SARS-CoV, which was also detected in SARS-CoV-2, was also linked to intracellular protein sorting and trafficking signals (12).

Our study showed the functional importance of conserved domains across the species barrier and revealed the possible roles of the 3a protein in the viral life cycle. The observations reported in this study merit experimental confirmation.

### Genome selection and annotation.

A total of 2,825 genomes, as of 5 April 2020, were downloaded from GISAID. Genomes were selected based on both completeness (>29,000 bp) and the high coverage option. Sequences were piped into Prokka v1.14.6 (13) with the “- -kingdom Viruses” flag enabled. ORF3a protein sequences were extracted, and amino acid sequences were further parsed for sequencing related artifacts, such as N characters and N strings. Based on these criteria, 2,782 genomes were selected for downstream analysis.

### Protein 3a alignment and detection of nonsynonymous amino acid changes.

The selected genomes were aligned using MAFFT v7.450 (14), and the multiple-sequence alignment (MSA) was viewed in Jalview v2.10.5 (15). Nonsynonymous amino acid variants were manually extracted from the amino acid MSA. The variant site locations were put into the Protein Variation Effect Analyzer, known as PROVEAN v1.1.3 (16). The selected −2.50 cutoff value represents a mean balanced accuracy (specificity versus sensitivity) of 78.17%.

### Domains, motifs, and membrane topology analysis.

The protein sequences for protein 3a in SARS-CoV and SARS-CoV-2 were downloaded from the Swiss model repository (17) with the UniProtKB accession numbers P59632 and P0DTC3/YP_009724391, respectively. Both sequences were aligned with MAFFT for direct comparison. Domain and motif scanning was performed through option 3 in the Web-based ScanProsite tool (18) available at https://prosite.expasy.org/scanprosite/. The consensus patterns for various domains were manually entered, and scans were run with high sensitivity to eliminate unwanted matches. Identified domains and motifs were manually inspected and identified through the sequence alignment and correlated with the various nonsynonymous amino acid variants.

Membrane topology of the ORF3a protein was detected using Protter (5). Default parameters were adopted for sequence-based topology visualization of SARS-CoV (NCBI accession number P59632), SARS-CoV-2 (UniProtKB accession number P0DTC3), RaTG13 (NCBI accession number QHR63301.1), pangolin CoV (EpiCoV accession number EPI_ISL_410721), and civet SARS (GenBank accession number AY572035) 3a proteins. The Protter server collects protein topology data from UniProt (19) or Phobius (20).

### Phylogenetic analysis.

Amino acid sequences of all 3a protein loci were aligned using MAFFT v7.450 (14). The alignments was passed through BMGE (21) to infer entropy values relevant to the phylogeny, with minimal reconstruction artifacts. A phylogenetic tree of aligned Orf3a amino acid sequences was constructed using FastME 2.0 (22), which builds an initial neighbor-joining (NJ) tree and improves topology by implementing the nearest-neighbor interchanges (NNIs) algorithm along with Felsenstein’s bootstrap iterations for branch support.
