# Supplementary material for: SARS-CoV-2 and ORF3a: Nonsynonymous Mutations, Functional Domains, and Viral Pathogenesis
Source: mSystems. 2020 May 5;5(3):e00266-20. doi: 10.1128/mSystems.00266-20 (PMC7205519; doi:10.1128/mSystems.00266-20)
Supplement: TABLE S1 [file mSystems.00266-20-st001.pdf]

Table S1. Protein domains analyzed in 3a protein. Protein domains in 3a protein of SARS-CoV (Accession # P59632) were compared to those of 3a protein in SARS-CoV-2 (Accession # P0DTC3), RaTG13 (Accession # QHR63301.1), Pangolin-CoV (Accession # EPI_ISL_410721) and SARS-Civets (Accession # AAU04650.1). Six protein domains were identified (I to VI). Domain I (not shown) includes a 15 aa signal peptide; aa = amino acid.

Domain	Position	Description	Domain/Motif in SARS-CoV-1	Domain/Motif in SARS-CoV-2	Conserved residues	Function of Domain in SARS-CoV	Reference	Non-synonymous mutations in SARS-CoV-2
**II**	**36-40**	TRAF3-binding motif	P**L**QAS	P**I**QAS	From position 36 to 40 in the aa sequence. Consensus sequence for the motif is **PxQx(T/S/D)**	Protein 3a associates with TRAF3 through the motif to recruit p105 and promote ubiquitination of ASC which provides the necessary signaling to activate NF-κB and the NLRP3 inflammasome	Siu et al, 2019	None
**III**	**91-133**	Ion channels (IC)	Point residues: Y91, H93 and Y109. The 127-133 domain is as follows in SARS-CoV is: **C**WL**C**WK**C** with 3 conserved cysteine residues	Point residues are conserved in SARS-CoV-2. The 127-133 domain in SARS-CoV-2: LWL**C**WK**C** with 2 conserved cysteine residues	Point aa residues in positions 91, 93 and 109. Also from position 127 to 133.	Evidence suggests that IC activity is not directly involved in virulence but rather in membrane rearrangement and virus non-lytic virus exit. Mutations in the point residues (91, 93, 109) abrogate IC activity. In contrast the 127-133 domain is associated with the localization of the IC to the membrane and activity.	Chan et al, 2009 Castaño-Rodriguez et al., 2018	Several mutations have been identified: H93Y (n=14, 0.5%), L127I (n=1; 0.04%), W128L (n=1; 0.04%), L129F (n=1; 0.04%), W131C (n=1, 0.04%). A mutation at the H93 position may be associated with loss of IC function. A W131C mutation adds a third cysteine residue to the domain, similar to SARS-CoV
		Cysteine rich domain	**C**133	**C**133	Position 81 to 160.	Cysteine residues associated with homodimerization of protein 3a (which is a tetramer). Mutation at the 6th Cysteine residue (C133) abrogates homodimerization of the protein	Lu et al., 2006	None
**IV**	**141-149**	Caveolin-binding motif	YDANYF**V**CW	YDANYF**L**CW	Position 141 to 149.	Potential interaction with caveolin-1 which can regulate virus uptake and trafficking of protein 3a to the plasma membrane or endomembranes (sites for virion assembly and release)	Padhan et al., 2007	One aa difference is present between SARS-CoV and SARS-CoV-2.
**V**	**160-163**	Yxxφ motif	YNSV	YNSV	Position 160-163.	The motif is essential for Golgi to plasma membrane transport. Mutation in this motif results in aggregation in the Golgi and associated with lipid droplets. Then the protein is degraded by lysosomal processes.	Minashki et al., 2014	None
**VI**	**171-173**	Di-acidic motif	**E**GD	**S**GD	Position 171-173. Consensus motif is **ExD**	The motif was thought to be a contributing factor for Golgi to plasma membrane. But deletion of this domain did not affect the transport of 3a protein to the membrane in SARS-CoV		In SARS-CoV-2 a E171S mutation disrupts the consensus for a di-acidic motif. Furthermore, a D173Y mutation (n=1, 0.04%) was also detected in one isolate completely disrupting the di-acidic motif.

1. Siu K-L, Yuen K-S, Castaño-Rodriguez C, Ye Z-W, Yeung M-L, Fung S-Y, Yuan S, Chan C-P, Yuen K-Y, Enjuanes L, Jin D-Y. 2019. Severe acute respiratory syndrome coronavirus ORF3a protein activates the NLRP3 inflammasome by promoting TRAF3-dependent ubiquitination of ASC. The FASEB Journal 33:8865–8877.

2. Chan C-M, Tsoi H, Chan W-M, Zhai S, Wong C-O, Yao X, Chan W-Y, Tsui SK-W, Chan HYE. 2009. The ion channel activity of the SARS-coronavirus 3a protein is linked to its pro-apoptotic function. Int J Biochem Cell Biol 41:2232–2239.

3. Castaño-Rodriguez C, Honrubia JM, Gutiérrez-Álvarez J, DeDiego ML, Nieto-Torres JL, Jimenez-Guardeño JM, Regla-Nava JA, Fernandez-Delgado R, Verdia-Báguena C, Queralt-Martín M, Kochan G, Perlman S, Aguilella VM, Sola I, Enjuanes L. 2018. Role of Severe Acute Respiratory Syndrome Coronavirus Viroporins E, 3a, and 8a in Replication and Pathogenesis. mBio 9.

4. Lu W, Zheng B-J, Xu K, Schwarz W, Du L, Wong CKL, Chen J, Duan S, Deubel V, Sun B. 2006. Severe acute respiratory syndrome-associated coronavirus 3a protein forms an ion channel and modulates virus release. Proc Natl Acad Sci USA 103:12540–12545.

5. Padhan K, Tanwar C, Hussain A, Hui PY, Lee MY, Cheung CY, Peiris JSM, Jameel S. 2007. Severe acute respiratory syndrome coronavirus Orf3a protein interacts with caveolin. J Gen Virol 88:3067–3077.

6. Minakshi R, Padhan K. 2014. The YXXΦ motif within the severe acute respiratory syndrome coronavirus (SARS-CoV) 3a protein is crucial for its intracellular transport. Virology Journal 11:75.
